# Preventive Cardio-Oncology: The Time Has Come

**DOI:** 10.3389/fcvm.2019.00187

**Published:** 2020-01-10

**Authors:** Sherry-Ann Brown

**Affiliations:** Department of Cardiovascular Diseases, Mayo Clinic, Rochester, MN, United States

**Keywords:** cardio-oncology, prevention, preventive cardiology, cancer, cancer survivorship, cardiac rehabilitation, cardiometabolic, risk factors

## Introduction

**Figure 1:**
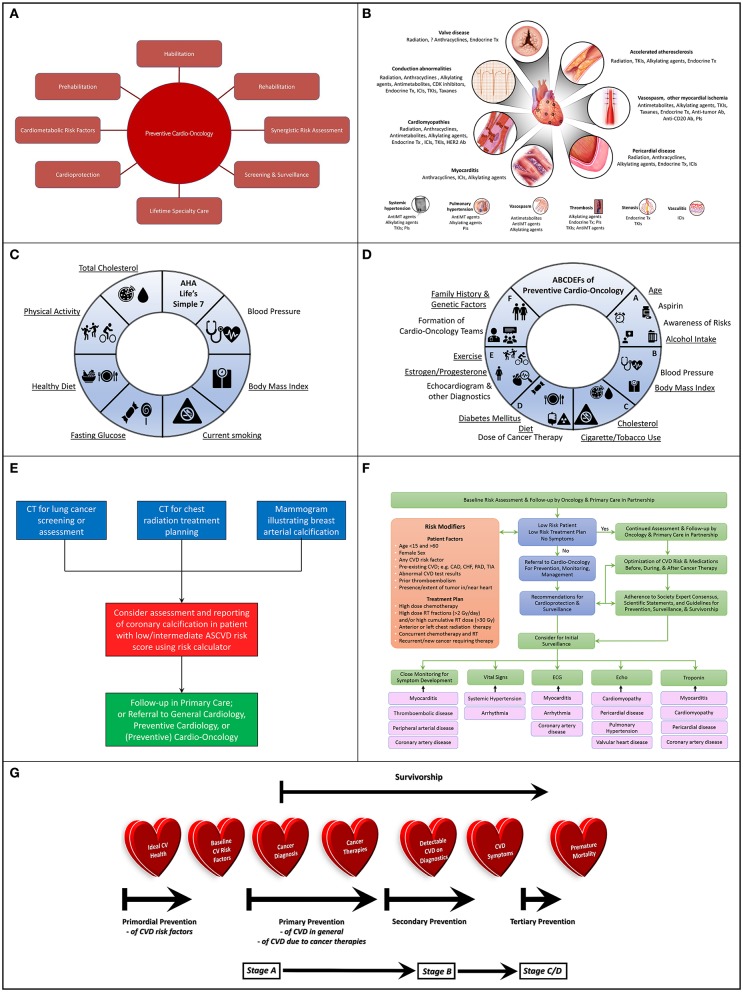
**(A)** Potential Scope of Preventive Cardio-Oncology: Preventive Cardio-Oncology can potentially consist of prehabilitation, habilitation, and rehabilitation, with synergistic risk assessment in cardiology and oncology screening tests, as well as optimization of cardiometabolic risk factors (pre-existing, or consequential from cancer/therapies), in addition to adequate cardioprotection in the setting of cancer therapy, with appropriate screening and surveillance guided by lifetime specialty care. **(B)** Cardiovascular Toxic Effects of Cancer Therapies: A wide spectrum of cancer therapeutics can injure or aggravate a variety of components of the cardiac (top) and vascular (bottom) system, knowledge of which can assist with vigilant monitoring, prevention if possible, and appropriate diagnosis if present. **(C)** American Heart Association (AHA) Life's Simple 7: Seven different domains can guide conceptualization and realization of Ideal Cardiovascular Health, recognizing specific factors (underlined) that underlie risk of development of both CVD and cancer. **(D)** ABCDEFs of Preventive Cardio-Oncology: A proposed ABCDEF approach to prevention of CVD and cancer in the general population and in those with a prior history of cancer addresses all seven factors in the AHA Life's Simple 7, as well as other characteristics that can contribute to CVD, cancer (new or recurrent), or both (underlined), with expansion of a previously published ABCDE algorithm (1, 2) to include Family History and Genetic Factors and Formation of Cardio-Oncology Teams to prevent or mitigate the impact of these multiple hits and risk factors relevant to the development of CV toxicities from cancer therapies. **(E)** Algorithm for Synergistic Screening in Preventive Cardio-Oncology: Patients presenting for screening or assessment for cancer could also be considered for simultaneous or subsequent CV screening and reporting, particularly if the initial imaging test is of the chest, with radiology teams scheduling or reading chest x-rays or chest CTs contacting patients' ordering clinician to consider CV assessment and reporting in tandem, or with ordering clinician teams (primary care, oncology, cardiology, cardio-oncology, preventive cardio-oncology) also requesting assessment and reporting of CV findings when ordering chest x-rays or CTs for patients at low/intermediate risk for CVD, especially for patients with any previous cancers who received radiation therapy or chemotherapy with drugs that associate with coronary artery atherosclerosis or thrombosis. **(F)** Algorithm for Risk Assessment and Follow-up: Initial assessment of patients in oncology can occur with the oncologist in partnership with primary care, with those patients at low risk continuing to be followed predominantly in oncology and primary care, while other patients can be referred to (preventive) cardio-oncology for recommendations on additional screening and preventive measures prior to, during, and after cancer therapies, recognizing that close monitoring for symptoms, abnormal vitals, or findings on lab testing, ECG, or Echo can help guide the need for more advanced testing related to potential cardiotoxicities; various specific cardiotoxicities may benefit most from particular initial monitoring methods (black arrows), e.g., myocarditis from ICIs may first manifest with symptoms with or without changes in the ECG or troponin (which can further be assessed with cardiac MRI), while VEGFI toxicity most commonly manifests as hypertension; high risk determinants are adapted from (3). **(G)** Preventive Cardio-Oncology Paradigm Shift: Efforts at prevention in Cardio-Oncology may need to be shifted earlier than traditionally implemented or currently considered; in typical Preventive Cardiology, primordial prevention targets the general population prior to development of risk factors for CVD, such as hypertension or diabetes; primordial prevention in Preventive Cardio-Oncology, if truly merging Preventive Cardiology and Cardio-Oncology, could benefit from maintaining this place of primordial prevention on the prevention spectrum; as such, indeed, primordial prevention would remain in the general population before cancer is diagnosed; at the time of cancer diagnosis, individuals in the general population would then become cancer survivors, with a focus on primary prevention for those with CV risk factors, then secondary prevention following cancer therapy, especially once structural heart disease is visible on imaging and manifested with signs or symptoms; with advanced CVD, tertiary prevention would be in effect, in hopes to optimize quality while delaying premature mortality. CAD, coronary artery disease; CHF, congestive heart failure; CV, cardiovascular; CVD, cardiovascular disease; Echo, echocardiogram; ECG, electrocardiogram; Gy, Gray; ICIs, immune checkpoint inhibitors; PAD, peripheral artery disease; RT, radiation therapy; TIA, transient ischemic attack; VEGFI, vascular endothelial growth factor inhibitor (a tyrosine kinase inhibitor).

The time has come for cardiovascular disease (CVD) prevention to play a more prominent role in cardio-oncology (Figure 1A). Cardio-oncology is an emerging subspecialty within internal medicine, and particularly cardiology, which involves the prevention and management of cardiovascular injury from cancer therapies (4–6). A small section of the field is dedicated to diagnosing and managing primary or secondary tumors to the heart (7). The majority of the field focuses on cardiotoxicity from radiotherapy, chemotherapy, and immunotherapy. While anthracyclines are the most commonly studied drugs and are frequently associated with cardiomyopathy, a myriad of novel cardiotoxic chemotherapeutic and immunotherapeutic drugs are continuously being developed in Oncology, with diverse cardiovascular (CV) effects (Figure 1B). Radiation to the chest can result in accelerated atherosclerosis, pericardial disease, valve disease, conduction abnormalities, and cardiomyopathies (8–10). Traditional chemotherapies, endocrine therapies, and targeted or immunotherapies can also associate with these cardiovascular toxicities in addition to myocarditis (3, 11–18), with valve disease possibly being no exception (19–22).

Various chemotherapies and targeted or immunologic therapies can also lead to peripheral vascular toxicities, such as systemic hypertension, pulmonary hypertension, thrombosis, stenosis, vasospasm, or vasculitis (3, 11–18) (Figure 1B). Systemic hypertension can further increase the risk of cardiovascular toxicity, as do all baseline CV risk factors (e.g., diabetes) and known CVD itself (11, 17, 23). Systemic hypertension occurs in more than one fourth of all individuals treated with vascular endothelial growth factor inhibitors (small molecule tyrosine kinase inhibitors), while all patients experience some increase in their blood pressure (24). These drugs also associate with cardiomyopathy, vasculopathy, coagulopathy, and nephropathy largely due to systemic endothelial dysfunction, reduced nitric oxide production, and destruction of the glomerular filtration barrier resulting from alteration of the balance between angiogenic and antiangiogenic, or vasoconstrictor and vasodilator, factors (24–26).

With all of this in mind, we should no longer direct the bulk of our efforts at intervention toward management of cardiovascular toxicities that have already occurred. It is time for us to swing the pendulum further in the direction of prevention of cardiovascular toxicities. Prevention can take different forms and can be pursued in different ways, with definition, scope, awareness, partnership, screening, surveillance, and intervention. Further, primordial (or health promotion), primary, secondary, and tertiary prevention may be defined somewhat differently in the traditional field of Preventive Cardiology (27, 28), than in a more recent proposal for consideration of prevention in cardio-oncology (20). It would be useful to reconcile these two slightly disparate paradigms, and attempt to continue to envision the potential scope of preventive efforts in cardio-oncology. Thus, as we advocate for moving from a traditionally reactive model to a more proactive model for prevention of cardiovascular toxicities in response to cancer therapies, we are entering the era of Preventive Cardio-Oncology. This concept is further introduced here, with a suggested near-future scope for the field (Figure 1A).

## Cancer Survivorship and Cardiometabolic Risk

As cancer therapies become more effective over time, the prevalence of cancer survivorship continues to rise. For 2019, almost 2 million new cancer diagnoses and just over 600,000 new cancer deaths are estimated; as cancer incidence continues to increase for most cancers, cancer mortality continues to decrease, leading to millions of cancer survivors each year (29). CVD remains a leading cause of death in cancer survivors (second only to cancer recurrence) (4, 20, 30, 31), and shares some risk factors with various cancers. For example, CVD and breast cancer have several risk factors in common, such as unhealthy Western diet, red or processed meat, physical inactivity or sedentary lifestyle, smoking, early menarche, and hormone replacement therapy (20) (Figures 1C,D, underlined). Yet, some factors affect risk differently for each disease. Premenopausal obesity and early menopause can increase CVD, while decreasing the risk of breast cancer; light to moderate intake of alcohol can attenuate cardiovascular risk, while increasing breast cancer risk (20). Given this reality, individuals diagnosed with breast cancer may already have baseline risk factors for CVD, as is also the case for individuals diagnosed with a variety of other cancer types. Overlapping risk factors for developing CVD or cancer (or both) (12, 16, 20, 32, 33) are underlined in Figures 1C,D.

Baseline cardiometabolic risk in individuals diagnosed with cancer can also conceivably increase even before beginning cancer therapies, because of cardiometabolic derangements or systemic inflammation due to the presence of the cancer itself (34, 35). Metabolic derangements and barriers to cardiometabolic health can also occur due to cancer therapies (4, 36–38). For example, chemotherapy can affect the vasculature and lead to initial diagnosis of, or aggravation of pre-existing, hypertension (12, 39). Radiation to the abdomen, brain, or whole body can affect the endocrine system resulting in abnormalities with hypothalamus-pituitary, thyroid, or pancreatic function (36, 40, 41), as well as hypertension, hyperlipidemia, and elevated waist circumference and levels of free fatty acids (40). Surgery to the brain can potentially also affect endocrine function, but appears to have very limited effect on cardiovascular risk, if any at all (42). A greater effect is seen when radiation is combined with surgery, and even greater so when chemotherapy also is added (42). In addition to direct physiological contributions to cardiovascular risk, cancer therapies can also have indirect effects. Fatigue, musculoskeletal discomfort, decrease in quality of life, or early menopause related to cancer therapies can limit individuals' ability to continue to pursue ideal cardiovascular health during therapy and can contribute to obesity and the metabolic syndrome (34, 36, 41). Thus, the CV impact of therapies for cancer (and potentially the presence of cancer itself) can superimpose on pre-existing CVD risk factors to worsen CV outcomes, which is described as the “multiple hit” hypothesis (23).

Given the co-existence of risk for cancer and CVD based on baseline factors, it has been suggested that joint screening for cancer and CVD should perhaps be pursued (32). This could include assessing for coronary artery calcification on chest CT scans initially ordered to screen for or evaluate known lung cancer or for radiation treatment planning in appropriate individuals, or following up on breast arterial calcification noted during mammography while screening for breast cancer (32, 43–47) (Figure 1E). When ordering chest x-rays or CTs for patients at low/intermediate risk for CVD, especially for patients with any previous cancers who received radiation therapy or chemotherapy with drugs that associate with coronary artery atherosclerosis or thrombosis, ordering clinician teams (primary care, oncology, cardiology, cardio-oncology, preventive cardio-oncology) could also request assessment and reporting of CV findings. Additionally, for patients with appointments for chest x-rays or chest CTs, radiology team schedulers and clinicians could contact patients' ordering clinicians to consider concurrent or subsequent CV assessment and reporting. A clinical trial (the BRAGATSTON Study) is currently in progress to provide low-cost automated quantification of coronary artery calcification on radiation planning chest CTs for CV risk prediction^1^. Similar studies could be pursued for chest CTs screening for or assessing cancers in the chest. Indeed, cardiovascular risk assessment in individuals diagnosed with cancer can uncover cardiometabolic risk factors out of proportion to the general population. CVD is more prevalent in individuals with cancer, compared to age-matched controls (48), and patients with cancer who develop CVD experience worse outcomes (31). Cardiovascular risk assessment should therefore be considered in tandem with screening for or managing various cancers.

Risk scores can play a role. In addition to the CVD risk scores used in the general population, such as the American Heart Association (AHA) and American College of Cardiology (ACC) pooled cohort atherosclerotic cardiovascular disease risk equations, there are also scores being developed for assessing CVD risk in individuals with a history of adult or childhood cancer (49, 50). Such risk scores may become invaluable in preventive cardio-oncology.

## Risk Mitigation

To mitigate cardiovascular risk in individuals with cancer, typical cardiovascular risk management should be pursued as in the general population with pharmacotherapy and lifestyle modification as appropriate (17, 51, 52), while recognizing that a history of cancer therapy increases overall cardiovascular risk and should inform the approach to lifestyle intervention (23) (proposed ABCDEF approach, Figure 1D).

### Pharmacologic Therapies and Oncologic Treatment Maneuvers in Prevention of Cardiomyopathy

Additional cardioprotective measures in the setting of cancer therapies should also be pursued as indicated (17). These can possibly include pharmacotherapy such as dexrazoxane, beta blockers, angiotensin converting enzyme inhibitors, angiotensin receptor blockers, statins, and other medications (53–55). Dose limitation or reduction for chemoradiation, as well as drug formulation and length of infusion for pharmacological cancer therapies can also be addressed (17, 55). Contemporary radiotherapy methods can also be adopted, with proton beam therapy, deep inspiration breath hold, prone imaging, and CT treatment planning, as well as dose limitation as appropriate (8–10, 55). Lifetime appropriate screening and surveillance would also be key (3, 4, 55). Meta-analyses and systematic reviews have suggested cardioprotective effects of beta blockers, angiotensin converting enzyme inhibitors, angiotensin receptor blockers, statins, dexrazoxane, and continuous infusion of a limited dose of liposomal doxorubicin, particularly for individuals with breast cancer receiving chemotherapy with anthracyclines, trastuzumab, or both (56–59). A single-center study determined deep inspiration breath hold as an effective method to reduce CV toxicity in patients with breast cancer (60). A multi-center randomized clinical trial is underway to assess benefits of proton vs. photon beam radiation therapy for individuals with non-metastatic breast cancer (61). Accordingly, cardioprotective pharmacotherapeutics and oncologic treatment maneuvers are currently most often used for patients with breast cancer who will undergo treatment with anthracyclines, trastuzumab, or both, especially those deemed to be high risk, e.g., based on chemotherapy dose, concurrent radiation, or pre-existing CVD, or those who have developed cardiotoxicity (17, 55) (Figure 1F). While further data needs to be obtained to strengthen recommendations for pharmacologic cardioprotection, studies so far support the likelihood of clinical utility in these patients.

### Pharmacologic Therapies in Optimization of Hypertension

For patients treated with vascular endothelial growth factors, antihypertensives usually provide sufficient control of blood pressure to allow for continuation of cancer therapy (14, 18). Most commonly, ACE inhibitors, ARBs, calcium channel blockers, and diuretics are used (18, 26, 62). ACE inhibitors have been thought to be preferred for concurrently impacting proteinuria, as well as release of endothelial nitric oxide and expression of plasminogen activator inhibitor-1 (63, 64). In fact, ACE inhibitors also associated with significant improvement in overall survival of patients with metastatic renal cell carcinoma (65). Of note, two recent retrospective studies were pursued using robust databases, with differences in study design, patient baseline characteristics, cancer type, and timing of use of antihypertensive therapy (26, 62). One study showed a greater extent of blood pressure control with ACE inhibitors and ARBS, compared to other antihypertensive drug classes (including calcium channel blockers and diuretics) (62). Conversely, the other recent study of patients treated with VEGF inhibitors identified blood pressure reductions most effectively associated with the use of calcium-channel blockers and potassium-sparing diuretics, compared to ACE inhibitors, ARBs, and other antihypertensive classes (26). Indeed, results from a preclinical study indicated a preference for nifedipine over captopril to achieve optimal control of severe blood pressure increases of 35–50 mm Hg (66). Thus, conclusions regarding inhibition of the renin-angiotensin-aldosterone system as the preferred method for managing hypertension in these patients have been divergent (67). Therefore, no clear broad recommendation can currently confidently be made for use of one antihypertensive class of medications over the other in all patients (64, 67). Care of the patient should continue to be individualized, and collaborations for patient care and further research in this area should persist (67). Nevertheless, a singular consensus remains: non-dihydropyridine calcium channel blockers specifically should be avoided, as these drugs inhibit the cytochrome P450 3A4 enzyme which metabolizes VEGFIs; use of non-dihydropyridine calcium channel blockers such as diltiazem or verapamil could lead to high levels of VEGFIs, potentially even further aggravating VEGFI-induced hypertension (18, 67, 68).

### Nutrition Counseling

Nutritional counseling could be personalized and re-assessed before, during, and after cancer therapy. Currently, dietitians have the option of training in Oncology as a nutrition subspecialty, which is particularly helpful for patients with cancer who develop malnutrition [estimated at 40% of all cancer patients, or >50% in patients with gastrointestinal or head and neck cancers (69, 70)]; dietitians can also become certified in Obesity and Weight Management^2^. Perhaps dieticians or nutritionists could be trained in preventive cardio-oncology and opt for certification in both Oncology Nutrition and Obesity And Weight Management, to merge philosophies from oncology and preventive cardiology in the context of individual patient nutrition needs. Indeed, studies in nutrition oncology encourage individualization of patients' nutritional care (71, 72), which may be a good fit as a component of Preventive Cardio-Oncology. A multicenter study indicated that a majority of clinicians in Oncology assessed patients for obesity and pursued weight management counseling during and after patients' cancer treatment, but did not refer patients for continued lifestyle intervention and support to optimize or maintain a healthy weight (73). Of note, a recent document providing recommendations and guidelines for obesity management in cancer highlights obesity as a risk factor for cancer development, recurrence, or outcomes, and cautions clinicians to be aware that obesity can sometimes mask acute/subacute malnutrition in individuals with cancer (74). Given the intricacies of obesity and nutrition for cancer patients and survivors, the American Society of Clinical Oncology has established a multifaceted initiative focused on education, awareness, research, policy, and advocacy for obesity and weight management in cancer patients and survivors (75). Perhaps partnerships among Oncology and Cardiology societies, including the American Society for Preventive Cardiology would be key in the advancement of Preventive Cardio-Oncology (75).

### Cardio-Oncology or Cardiovascular Oncology Prehabilitation, Habilitation, Rehabilitation

Incorporation of various methodologies in typical cardiac rehabilitation could also have utility for secondary prevention (76), and possibly also primary prevention, of cardiovascular toxicity from a variety of cancer therapies. Some have suggested a multidimensional system consisting of cardiovascular oncology prehabilitation (30, 77, 78), habilitation (30, 79, 80), and rehabilitation (30, 78, 81), to optimize cardiovascular health before, during, and after cancer therapies, respectively. Prehabilitation focuses on optimizing baseline cardiovascular health and cardiopulmonary fitness prior to initiation of cancer therapies (30, 77, 78). Habilitation refers to ongoing optimization of cardiovascular health and cardiopulmonary fitness while patients are undergoing cancer therapies (30, 79, 80). Rehabilitation refers to efforts to recover cardiopulmonary function after completion of cancer therapies (30, 78, 81).

### Potential Challenges in Cardio-Oncology Prehabilitation, Habilitation, Rehabilitation

There is often little time from diagnosis of cancer to initiation of chemotherapy, which can limit the time window for pursuing cardio-oncology prehabilitation in the cardiology community. Nevertheless in the oncology community, timely calls to action for developing high-quality cancer rehabilitation programs propose prospective models that actually begin upon cancer diagnosis and continue throughout cancer therapy then for a lifetime (82, 83), which could also be termed cancer prehabilitation (before therapy), habilitation (during therapy), and rehabilitation (after therapy). These multidisciplinary programs aim to optimize physical and psychosocial health and function. The models include establishment of baseline functioning prior to cancer therapies. This can include tests such as the 6-min walk or Get-Up-And-Go, or formal fitness assessment as in the general population prior to exercise training (84). This can help assure both patients and clinicians of the safety of pursuing exercise training (84). Fitness testing as part of baseline assessment can also identify potential physical vulnerabilities that may limit or modify exercise regimens (84). Pre-existing conditions that may associate with high risk for cardiovascular and other toxicities can be identified and addressed; exercise level, mobility, strength, and gait, among other parameters, are objectively assessed. Ongoing surveillance subsequently occurs during and after therapy, with referrals made for exercise, nutrition, and weight management. With further collaboration between the cardiology and oncology communities, efforts could be combined to establish and pool resources and protocols for comprehensive multidimensional cardio-oncology programs pursued before (prehabilitation), during (habilitation), or after (rehabilitation) cancer therapy.

Cardio-oncology habilitation can be challenging for some patients, due to physical or psychosocial effects of cancer therapy. Studies have shown reduction in physical activity levels in cancer patients following diagnosis and during therapy (85–87). In fact, patients have traditionally often been advised to limit their physical activity during therapy, particularly for those who are older or experience fatigue or side effects of cancer therapy (82). The most frequent symptoms in cancer patients include fatigue and peripheral neuropathy. Of note, aerobic, resistance, and strength training have been shown to improve fatigue; strength and balance training also improve fatigue (88).

It should be recognized that patients often maintain lower physical activity levels even after completion of cancer therapies, and generally do not return to their pretreatment levels of physical activity (85–87). In spite of a myriad of guidelines and recommendations from various societies regarding the safety and efficacy of exercise (75, 89, 90)^3^, ~70% of cancer survivors do not meet recommendations for physical activity in cancer survivors (91, 92). It should be noted too that adverse effects of cancer therapies extend to the entire cardiovascular and skeletal muscle axis (76). As a result, cardiorespiratory fitness (CRF) declines are in part due to impairment of components of the cardiovascular and respiratory muscle system. Thus, survivors may have impairments in mobility and function beyond the general population (93). For example, CRF in survivors can be 30% lower than in age-matched healthy yet sedentary control individuals (94), and as stated often does not recover following cancer therapy (94–96). However, physical function and cardiorespiratory fitness can both improve with structured exercise regimens in cancer rehabilitation (85–87, 97). Additionally and importantly, post-treatment exercise reduces the risk of mortality (98–102).

In one established program, oncology rehabilitation is modeled based on cardiac rehabilitation and is available to all cancer survivors, with no restrictions on length of time since diagnosis^4^ (103). Evaluation by a physical therapist clears patients for safe and effective exercise therapy. Physical activity is tailored to each patient's capabilities and limitations. The initial focus is on brisk walking and resistance training. More than 500 individuals have gone through the program, with significant improvements in peak oxygen consumption and the 6-min walk test, as well as upper and lower extremity strength. Patients start slowly and are encouraged to aim for similar recommended physical activity levels as the general population^5^ (89, 104). The system is similar to recommendations in the AHA statement on Cardio-Oncology rehabilitation, which also includes a suggestion for “opt-out” referrals to maximize utilization (76).

Survivors find it quite helpful to have supervised exercise in the context of social support, encouragement, motivation, and accountability from other patients or from oncologic/cardiac rehabilitation staff. The convenience of having cardiac rehabilitation facilities close to and affiliated with the medical institution that hosts the cancer center may help cancer patients and survivors embrace cardio-oncology rehabilitation programs in the post-adjuvant period (i.e., after completion of cancer therapies). This should also apply to cardio-oncology prehabilitation (before cancer therapies; neoadjuvant?) and habilitation (while undergoing cancer therapies; adjuvant) programs. Studies show that cardiovascular fitness is preserved or improved if exercise training is initiated prior to completion of cancer therapy, rather than waiting to recover cardiopulmonary function after completion of cancer therapy (105, 106). This provides support for the benefit of habilitation even over rehabilitation. If programs initiate exercise training before the first dose of cancer therapy, this points toward the potential for prehabilitation.

In the general population, outpatient cardiac rehabilitation has low utilization rates, although there is potential to decrease morbidity and mortality by ~25% (107). Women in particular face a myriad of barriers in cardiac rehabilitation (108, 109), and large numbers of women are not referred at all (108, 109). High intensity interval training (HIIT) may be among practical solutions to meet women's needs in cardiac rehabilitation (108). HIIT provides more effective and efficient regimens than typical exercise programs, at all stages of prevention (104). HIIT has been shown to improve cardiovascular health components such as fitness (110, 111), body fat percentage (111, 112), waist circumference (112), insulin resistance (111), blood glucose levels (113), blood pressure (113), and lipids (111, 112) in the general population, as well as those with a history of cardiac disease (114, 115) and in patients with a history of cancer (116). Thus, HIIT should play role in Cardio-Oncology prehabilitation, habilitation, and rehabilitation. However, HIIT will not overcome all barriers. Besides time limitations, accessibility, education level, multiple comorbidities, language, social support, and family responsibilities also play a role. Automated referrals or “opt-out” methods may improve referral and participation of women, as may enrollment assistance, incentives, and home-based exercise regimens (109). The remaining barriers of health literacy, multiple comorbidities, language, family responsibilities, and side effects from cancer therapy will also need to be addressed.

Outpatient cardiac rehabilitation utilization in the general population is also particularly limited in rural communities, as well as in those with limited education, lower socioeconomic status, or older age (107). Research is therefore needed to continue to assess the feasibility and effectiveness of these proposed cardio-oncology prehabilitation, habilitation, and rehabilitation programs in academic and community centers or in patients' homes preferably with virtual exercise trainers or accountability groups. Innovative approaches with telemedicine, mobile health, and remote monitoring are being evaluated as potentially cost-effective means of delivery of these structured programs.^6^

### Cardiopulmonary Stress Testing in Prehabilitation, Habilitation, Rehabilitation

It would also be ideal for cardiopulmonary stress testing to be incorporated to assess cardiopulmonary functional status before, during, or after cancer therapies. This could potentially be helpful for preventive counseling and subsequent management for any abnormalities in cardiopulmonary function, given that various cancer therapies can injure the heart and lungs and lead to reduced cardiopulmonary function and fitness (94, 95, 117–119), which associates with increased risk of long-term cardiovascular toxicities, cardiopulmonary symptoms, and mortality (120, 121).

### Rehabilitation Too Late?

Currently, the AHA has issued a Scientific Statement endorsing cardio-oncologic rehabilitation, including cardiopulmonary stress testing after patients have completed cancer therapy, particularly for those who received high doses of cardiotoxic chemotherapy or radiation and who have known cardiopulmonary symptoms, CVD, or untreated CVD risk factors (76). The statement is supported by various studies suggesting the benefit of structured exercise therapy for improving cardiovascular health and cardiopulmonary or fitness for cancer survivors (30, 81, 97, 122). In turn, cardiopulmonary fitness associates with lower levels of short-term and long-term cardiovascular and other toxicities, as well as a lower risk of mortality in individuals with cancer (94, 120, 121). Studies also indicate benefits of exercise prior to initiation of or during cancer therapy (30, 116, 123). The statement also encourages optimization of patient evaluation, nutrition and dietary counseling, obesity management, blood pressure and lipids management, diabetes, tobacco cessation, and management of psychosocial contributing factors (76). Additionally, the AHA statement encourages continued research to help determine best practices for implementation of cardio-oncology rehabilitation and determine of clinical indication and feasibility to support reimbursement.

Perhaps once rehabilitation becomes more established, we can then also strategize and implement cardio-oncology prehabilitation and habilitation. Yet, will it be too late to wait? Should we primarily focus our efforts on rehabilitation after cancer therapies, or should we turn our attention to habilitation during cancer therapies while patients are undergoing cycles of chemotherapy and radiation before or after surgery? In fact, should we start sooner? Should we advocate for initiating cardio-oncology prehabilitation before patients undergo chemotherapy, radiation, or surgery? In the overall surgical world, exercise or nutritional prehabilitation (initiated before surgery) seems to improve outcomes and lower healthcare costs compared to waiting for rehabilitation (initiated after surgery) alone (124–137). Clinical trials are underway to further define the prehabilitation period (before surgical therapy) as optimal for fitness intervention (138–140). Data for cardio-oncology prehabiltiation is also promising. Most recently, a study suggested that long-term survivors of breast cancer who had higher levels of physical activity (and presumably fitness) prior to their cancer diagnosis demonstrated a graded reduction in subsequent CV outcomes (e.g., coronary artery disease, heart failure, peripheral artery disease, cardiovascular death), compared to their counterparts with lower levels of physical activity pre-diagnosis (123). Does this also provide indirect support for stronger consideration of primordial and primary prevention in cardio-oncology (Figure 1G)? Primordial prevention focuses on preventing development of CVD risk factors such as hypertension, hyperlipidemia, or obesity. Primary prevention focuses on managing existing CVD risk factors such as hypertension, hyperlipidemia, obesity, or diabetes, in efforts to prevent development of CVD itself. If through primordial and primary prevention in the general population we can optimize ideal cardiovascular health (e.g., based on the AHA Life's Simple 7) before cancer diagnoses occur, then perhaps when individuals from among the general population are diagnosed with cancer and become cancer survivors, those with greater CV health may already be ahead of the curve. The AHA Life's Simple 7 campaign encourages optimization of seven modifiable cardiovascular risk factors, specifically diet, physical activity, weight, smoking, hypertension, hyperlipidemia, and diabetes (141, 142) (Figure 1C).

## Paradigm Shift

In Preventive Cardiology, the prevention spectrum includes primordial, primary, secondary (and often tertiary) components. For Cardio-Oncology, a prevention timeline has been suggested in a recent AHA Scientific Statement on the intersection of CVD and breast cancer (20), specifically for the development of cardiovascular toxicity from cancer therapies, regardless of the presence of baseline cardiovascular health and risk factors. In this timeline, primordial prevention of cardiotoxicity would begin upon cancer diagnosis before cancer therapies are administered, since administration of cancer therapies is the key risk factor for development of cardiotoxicity from cancer therapies. Primary prevention would then begin after receiving cancer therapies as the main risk factor for cardiotoxicity. Secondary prevention would begin upon diagnosis of structural heart disease, such as decline in left ventricular systolic function, thought to be due to administration of cancer therapy. Treatment of subsequently developed CVD could then be considered tertiary prevention in this model.

In pursuit of preventive cardio-oncology, we will need to determine how to align the typical paradigm in preventive cardiology with the timeline proposed in the recent scientific statement. Perhaps the two paradigms could be reconciled as follows (Figure 1G). Ideal cardiovascular health based largely on the AHA Life's Simple 7 would be the initial baseline in the general population, from among which cancer survivors will ultimately be diagnosed. This ideal baseline would include optimal parameters for diet, physical activity, obesity, smoking, hypertension, hyperlipidemia, and diabetes, as well as nontraditional cardiovascular risk factors. Primordial prevention of CVD in the general population would limit or avoid development of CVD risk factors (e.g., hypertension). If baseline CVD risk factors develop (e.g., hypertension or hyperlipidemia), then primary prevention would ensue in two different and complementary categories. The first category would be typical primary prevention of CVD in the general population, optimizing management of existing traditional and nontraditional cardiovascular risk factors. The second category would add cardioprotective measures if indicated for those who have been diagnosed with cancer and are deemed to be high risk for developing cardiotoxicity (Figures 1F,G). Such measures may include utilization of beta blockers, angiotensin converting enzyme inhibitors, angiotensin receptor blockers, statins, dexrazoxane, continuous infusion of a limited dose of liposomal doxorubicin, and potentially proton vs. photon beam therapy, and so on.

Once cancer therapies are administered, subclinical cardiovascular injury undetected by standard diagnostic testing may be presumed, since studies have shown microscopic evidence of cardiovascular injury (preclinical) and development of macroscopic CVD (clinical) with even low doses of cancer therapy (143–150). Thus, one could argue that secondary prevention of cardiovascular injury could start following administration of any dose of cancer therapies. However, undetected subclinical injury could be considered Stage A akin to the corresponding ACC stage of heart failure (143), consistent with the presence of risk factors for CVD without overt evidence of macroscopic CVD detected on standard diagnostic tests (151, 152). Once detection of CVD on diagnostic testing occurs, then this would be classified as Stage B, if no symptoms have yet developed, with continuation of secondary prevention. Traditionally, change in left ventricular ejection fraction has been most commonly used to identify cardiotoxicity, heralding Stage B/C CVD; more recently, studies are underway (including an international multicenter prospective randomized controlled trial) to assess the efficacy of global longitudinal strain as a more sensitive and earlier echocardiographic marker of cardiovascular injury in Stage B (153, 154). Once cardiovascular symptoms develop, then Stage C CVD would be diagnosed. Tertiary prevention would be initiated at this point, to avoid complications or further events, or progression of CVD to Stage D, or eventually premature mortality (Figure 1G).

## Partnerships for Monitoring, Surveillance, and Intervention in Prevention

It is interesting to consider who should be responsible for CV screening, monitoring, and treatment in Preventive Cardio-Oncology, in the short-term and long-term, before, during, and after cancer therapies. There are several excellent algorithms in literature to guide the flow of patient care in Cardio-Oncology (3, 11, 14, 17, 18, 155–158). Most of these algorithms focus on the relationships among the cardiologist, oncologist, hematologist, and patient, or on steps to follow regarding surveillance for or management of left ventricular dysfunction or other cardiovascular toxicities, or monitoring recommendations for patients being treated with particular cancer therapies. Very few if any of the algorithms explicitly incorporate partnership with patients' primary care providers, and if so, where in the algorithm their input would be present. It goes without saying that patients' primary care providers are understood to be part of their care at all stages. Nevertheless, it may be helpful to clearly include primary care expertise in Preventive Cardio-Oncology algorithms. In Figure 1F, for example, baseline screening and follow-up for all patients in Cardio-Oncology traditionally begins in Oncology (or Hematology), and this should be in partnership with our colleagues in Primary Care. Involving Primary Care clinicians from early on may help streamline long-term follow-up of cancer patients and survivors. A study revealed the prevalent interest and practice of Primary Care clinicians in optimal long-term care of cancer survivors, juxtaposed with their concerns about limited information received regarding patients' detailed treatment plan and survivorship care recommendations (159). Primary Care clinicians also appear to generally desire more guidance, communication, and training to best manage shared care and delegation of responsibility for complications that arise for cancer survivors (159).

Perhaps in Preventive Cardio-Oncology, we can establish distinct methods and training to help guide assessment and follow-up of patients in the context of their (Preventive) Cardio-Oncology team (in F portion of ABCDEF, Figure 1D). At baseline, asymptomatic patients with low risk medical history and low risk treatment plans could continue to be followed closely in Oncology and Primary Care, with a goal of optimizing CVD risk and medications before, during, and after cancer therapy, guided by Cardiology if needed (Figure 1F). In fact, if at any point before, during, or after cancer therapy, patients are symptomatic or are not deemed to be at low risk based on their medical history or treatment plan, these patients should be evaluated to determine the best roles for prevention and management at that moment in their Cardio-Oncology care. A role for Preventive Cardio-Oncology would include providing recommendations for cardioprotection, screening, and surveillance, especially prior to initiation of cancer therapy. All care decisions for patients should be guided by individualization of care, with adherence to any available society expert consensus or scientific statements, or guidelines for prevention, surveillance, and survivorship, as well as management (12, 16, 18, 20, 55, 76).

Minimal surveillance prior to most if not all cancer therapies should include close monitoring for development of symptoms, as well as vital signs and ECG, and often echocardiogram, and troponin (Figure 1F). On subsequent testing, these modalities could most often capture initial signs of cardiovascular toxicity. Various forms of early toxicity may be more likely to show up on some modalities than others (black arrows, Figure 1F). For example, most groups recommend ECG and troponin for monitoring of early toxicity from immune checkpoint inhibitors, of course along with observation for symptoms and assessment of vitals; with escalation to cardiac MRI if suspicion for myocarditis persists, or endomyocardial biopsy, if indicated, in conjunction with Cardio-Oncology consultation and management (13, 160–163). Weekly monitoring of troponin may be pursued for the first 6 weeks, given that aggressive and often fulminant myocarditis typically occurs at a median of 30 days from initiation of therapy (13, 164). However, it should be recognized that troponin levels, echocardiography, or electrocardiography can be normal in patients presenting with myocarditis (162); a high index of suspicion is paramount for any non-specific symptoms or signs noted in patients treated with immunotherapy. As another example, tyrosine kinase inhibitors such as vascular endothelial growth factor inhibitors most commonly aggravate hypertension; accordingly, monitoring and close management of vital signs is of utmost importance in patients being treated with these drugs (165, 166). Weekly monitoring of blood pressure (goal <140/90 mm Hg) for the first 4–6 weeks is recommended, and subsequently every 2–3 weeks until completion of therapy, as systemic hypertension as a form of CV toxicity usually manifests within 4 weeks (14, 18, 26, 62, 67, 167, 168). For patients receiving anthracyclines, an echocardiogram (or cardiac MRI or MUGA if echocardiography is not available or technically feasible) is usually pursued prior to initiation of therapy and within 6–12 months of completion of therapy particularly in high-risk patients, or sooner if concerning CV signs or symptoms arise for any patient during therapy (17, 55). For treatment with trastuzumab, an echocardiogram is commonly pursued at baseline and at 3, 6, and 9 months and often indefinitely while on therapy, although the timing of surveillance remains debatable^7^ (55). Recognizing the need for differential monitoring of patients based on their treatment regimen is critical to excellent team-based care in (Preventive) Cardio-Oncology, particularly since some drugs are more cardiotoxic than others (Figure 1B).

Following therapy, surveillance and screening in Preventive Cardio-Oncology can be guided by algorithms based on individual patients' treatment plans. All patients could pursue an ABCDEF approach (Figure 1D), which expands on the established ABCDE approach (1, 2), to optimize CV health. For asymptomatic patients with normal cardiac function following therapy with anthracyclines or trastuzumab, while no strict recommendations are available for frequency and duration of echocardiographic or biomarker surveillance, management of CV risk factors should be pursued lifelong (55). Those treated with vasotoxic drugs with persistent cardiovascular risk after therapy (e.g., cisplatin, nilotinib) could consider imaging formal screening tests for peripheral artery disease every 1–2 years and non-invasive stress testing every 5 years (3). Non-invasive stress testing could also be considered every 5 years, along with a concomitant transthoracic echocardiogram, for those who received radiation therapy (3, 158). Most recently, a study illustrated a strong correlation between coronary artery radiation exposure and subsequent segmental coronary artery calcification scores (169). New coronary artery calcification was noted in some patients with breast cancer, lung cancer, lymphoma, or myeloma at a median of 32 months after radiation therapy. Recommendations could be developed for using coronary artery calcification for surveillance in cancer survivors starting 2–3 years after radiation therapy, for those individuals who would otherwise be at low/intermediate CVD risk. The current ACC/AHA prevention guidelines suggest consideration of statin therapy for any CAC > 0 (51). Perhaps this recommendation could also be considered for cancer survivors, who are at an even greater risk for developing CVD than the general population. Perhaps in the future prophylactic statin initiation may be considered for those with higher levels of mean heart dose or coronary artery radiation exposure, even in the absence of (1) known CAC score or (2) CAC score > 0. In the current prevention guidelines, a CAC score of 0 does not preclude statin therapy, if individuals have a personal history of diabetes or cigarette smoking, or a family history of premature coronary heart disease (51). Preventive Cardio-Oncology recommendations could include a history of treatment with radiation therapy or vasotoxic drugs with persistent risk (e.g., cisplatin, nilotinib) in this list of risk modifiers.

## Discussion

We can no longer wait to intensify our focus on prevention in cardio-oncology, as the number of survivors of childhood and adult cancers approaches 17 million in 2019 and is projected at ~22 million by 2030^8^. The presence of baseline cardiovascular risk factors raises CVD risk after cancer therapies, but at a higher rate than in the general population due to cardiotoxic effects of cancer therapies. Risk mitigation strategies in the general population are also applied in cardio-oncology, while recognizing the higher risk. Cardioprotection specific to cardio-oncology (e.g., dexrazoxane) is also potentially used, along with appropriate screening and surveillance. A paradigm shift may be beneficial, to initiate preventive efforts sooner than currently considered.

One in three men and women will develop cancer over the course of their lifetime^9^, and many of these individuals will become long-term cancer survivors. A greater emphasis on encouraging and facilitating healthy behaviors, screening and surveillance, and other methods of cardioprotection will be needed for cancer survivors, as well as novel methods of identifying those at greatest CVD risk (170). Cardiopulmonary function testing and cardio-oncology prehabilitation, habilitation, and rehabilitation will likely help promote efforts at achieving and maintaining ideal cardiovascular health characterized by optimization of Life's Simple 7, along with non-modifiable and nontraditional risk factors. These will need to be applied for survivors of adult and childhood cancers, with improved care transition when children and adolescents become adults. Lifetime access to specialty care will need to continually be established, as particularly desired by childhood cancer survivors (171, 172). Disparities in the medically underserved and in ethnic minorities will also need to be addressed (170), particularly as efforts in precision cardio-oncology and innovation in cardio-oncology unfold.

Research should continue in all of these arenas, to help inform best practices for prevention in cardio-oncology. Several clinical trials are underway to assess CV risk factors and profiles in individuals with a variety of cancer types being treated with a myriad of chemotherapies and endocrine therapies at various stages following diagnosis^10^. These studies are utilizing traditional or innovative (e.g., Clinical Informatics using the electronic health records) methods to assess cardiovascular risk profiles in the short-term or long-term for patients with breast, endometrial, prostate, testicular, or colorectal carcinoma, or Hodgkin's or Non-Hodgkin's lymphoma, or other cancers treated with endocrine therapies or other forms of cancer therapy. Dozens of clinical trials are also ongoing to assess potential benefits and cost-effectiveness of preventive efforts with pharmacotherapeutics (e.g., statins, ACE inhibitors, beta blockers) before, during, or after cancer therapies, and nutrition and exercise initiated before, during, or after cancer therapies (for cardio-oncology prehabilitation, habilitation, or rehabilitation)^11^. Pharmacologic cardioprotection will likely continue to be the most feasible intervention to institute, for high risk patients prior to initiation of cancer therapies. Prehabilitation with nutrition and exercise for those diagnosed with cancer may have a limited timeframe for initiation. Nevertheless, it may be prudent to counsel those diagnosed with cancer about nutrition and exercise benefits even prior to starting canscer therapies. Valuable efforts in prehabilitation may indeed also be to partner with Preventive Cardiologists and Primary Care clinicians to encourage health promotion and synergistic surveillance and screening in the general population before cancer diagnoses are made. Perhaps with a somewhat thorough approach to preventive cardio-oncology, we may be able to prevent some injury, protect more hearts, and facilitate cancer remission or cure, with a goal of greater quality and quantity of life.

## Author Contributions

S-AB conceived of designed, wrote, revised, and approved the submitted manuscript.

### Conflict of Interest

The author declares that the research was conducted in the absence of any commercial or financial relationships that could be construed as a potential conflict of interest.

## Footnotes

^1^http://www.clinicaltrials.gov

^2^https://www.cdrnet.org/certifications/board-certification-as-a-specialist-in-oncology-nutrition

^3^https://www.nccn.org/professionals/physician_gls/pdf/survivorship.pdf

^4^https://professional.heart.org/professional/ScienceNews/UCM_503836_Exercise-focused-Rehabilitation-for-Cancer-Survivors-Creating-a-CORE-Component.jsp

^5^https://professional.heart.org/professional/ScienceNews/UCM_503836_Exercise-focused-Rehabilitation-for-Cancer-Survivors-Creating-a-CORE-Component.jsp

^6^https://professional.heart.org/professional/ScienceNews/UCM_503836_Exercise-focused-Rehabilitation-for-Cancer-Survivors-Creating-a-CORE-Component.jsp (82).

^7^National Comprehensive Cancer Network, NCCN Clinical Practice Guidelines in Oncology. Breast Cancer. Version 3.2014. https://jnccn.org/abstract/journals/jnccn/12/4/article-p542.xml

^8^National Cancer Institute Office of Cancer Survivorship. Statistics: National Cancer Institute; 2019 [Available from: https://cancercontrol.cancer.gov/ocs/statistics/index.html]

^9^The American Cancer Society. Lifetime Risk of Developing or Dying From Cancer: American Cancer Society; 2018. Available from: https://www.cancer.org/cancer/cancer-basics/lifetime-probability-of-developing-or-dying-from-cancer.html

^10^Examples of trials at http://www.clinicaltrials.gov, https://clinicaltrials.gov/ct2/show/NCT01080170, https://clinicaltrials.gov/ct2/show/NCT00885612, https://clinicaltrials.gov/ct2/show/NCT00705094, https://clinicaltrials.gov/ct2/show/NCT00688532, https://clinicaltrials.gov/ct2/show/NCT03935282

^11^Examples of trials at http://www.clinicaltrials.gov, https://clinicaltrials.gov/ct2/show/NCT02842658, https://clinicaltrials.gov/ct2/show/NCT02969577, https://clinicaltrials.gov/ct2/show/NCT03964142, https://clinicaltrials.gov/ct2/show/NCT02454777, https://clinicaltrials.gov/ct2/show/NCT03131024, https://clinicaltrials.gov/ct2/show/NCT03277898, https://clinicaltrials.gov/ct2/show/NCT03334071, https://clinicaltrials.gov/ct2/show/NCT03748550, https://clinicaltrials.gov/ct2/show/NCT02471053, https://clinicaltrials.gov/ct2/show/NCT03748550, https://clinicaltrials.gov/ct2/show/NCT03975491
